# Toolkit for acoustic–phonetic analysis of naturalistic speech data

**DOI:** 10.3758/s13428-026-03174-y

**Published:** 2026-09-09

**Authors:** Ethan Kutlu, Emerson Peters, Ciara Tapanes, Sarmad Chandio, Osama Khalid

**Affiliations:** 1 Department of Communication Sciences and Disorders, Department of Psychological and Brain Sciences, Department of Linguistics, University of Iowa, Iowa City, IA USA; 2 Department of Speech & Hearing Sciences, University of Washington, Seattle, WA USA; 3 Department of Spanish and Portuguese, University of Iowa, Iowa City, IA USA; 4 Department of Computer Science, University of Iowa, Iowa City, IA USA

**Keywords:** Acoustic–phonetic analysis, Computational approaches, Speaker diarization, Naturalistic data, Natural language processing, Speech processing, Voice onset time, Spectral moment

## Abstract

A major limitation in the speech sciences is access to naturalistic data in experimental settings and the difficulty of translating laboratory designs to real-world contexts. Researchers studying speech production or perception often rely on in-lab recordings, which constrain the sociolinguistic contexts examined and limit ecological validity. The Toolkit for Acoustic–Phonetic Analysis (TAPA) is an open-source pipeline that automates the acquisition, transcription, speaker diarization, forced alignment, and per-segment acoustic analysis of naturalistic, single/multi-speaker audio. The current release supports vowel formant extraction, stop voice onset time, and fricative spectral moments. We demonstrate TAPA on the 2016 U.S. presidential debate, extracting nearly 33,000 segments from a 90-min recording, and validate each measurement type against hand-coded annotation. Vowel formant agreement with expert measurements was high (F1 *r* = 0.89, F2 *r* = 0.87). Stop VOT showed reliable aggregate means but poor per-token agreement (*r* =  − 0.04) because of a training–deployment mismatch in the neural VOT classifier. Fricative spectral standard deviation agreed strongly with hand-coded values overall (*r* = 0.80), and center of gravity agreed strongly for sibilants (/s/ *r* = 0.87, /ʃ/ *r* = 0.94), while non-sibilant moments diverged systematically. These findings suggest that TAPA can be used to increase access to naturalistic speech data and speed up the processing timeline with experts’ supervision.

## Introduction

Research on spoken language traditionally relies on controlled laboratory speech. These stimuli are generally carefully crafted word- or sentence-level productions by participants in a lab setting. Many initiatives helped speech scientists immensely through this approach in testing theories and frameworks for decades (Bradlow, n.d.; Van Engen et al., 2010). These initiatives, combined with in-lab testing, allow precise control over variables and facilitate repeatability and replicability. However, the need for naturalistic data has been increasingly visible in speech sciences and language science research. Ample evidence now shows that listeners and speakers dynamically affect each other in everyday conversations (Babel, 2022; Docherty & Foulkes, 2014; Hay & Drager, 2007) (also see Ivanova et al., 2020). This process has also been studied widely in psycholinguistic literature through in-lab testing where participants show adaptation to input (Alexander & Nygaard, 2019; Aoki & Zellou, 2023; Babel, 2022; Bent et al., 2009; Bradlow & Bent, 2008; Clarke & Garrett, 2004; Xie et al., 2017). Such influences longitudinally shift listeners’ and speakers’ perception and production as well as their language learning and processing strategies (Bice & Kroll, 2019; Gu et al., 2026; Hanulíková & Levy, 2025; Kutlu et al., 2024; Syrett et al., 2024; Theodore & Miller, 2010; Theodore & Monto, 2019; Wigdorowitz, 2024). Therefore, our theories and frameworks require naturalistic data that occurs in everyday life speech instances. Various psycholinguistic and experimental sociolinguistic studies have already shown the immediate impact of more naturalistic data on speech models (Afkir & Zellou, 2026; Antetomaso et al., 2017; McMurray et al., 2023; Xie et al., 2023).

While the need for naturalistic data is increasing, naturalistic production data still remain scarce due to the laborious nature of data collection and numerous logistical and methodological barriers (see, for example, Johnson, 2021; Johnson & Babel, 2024, where the creation of the speech corpus and its laborious nature were highlighted). These obstacles not only hinder the gathering of high-quality speech corpora but also limit the scope of questions researchers can confidently answer about real-world language use. Importantly, these limitations create inequalities in research practices, as researchers who spend considerable time collecting and analyzing naturalistic speech data (e.g., sociolinguists, sociophoneticians) are also kept on the periphery of experimental speech science (Tripp & Munson, 2023). This work aims to bridge this gap by leveraging computational tools to enable a new, fully open-source, open-access means of production data collection and analysis. In doing so, we address a core need in the field: increasing access to robust naturalistic speech data for language science researchers. It is integral to note that this toolkit is not intended to replace researchers but to aid them by reducing the time spent on the acquisition and processing naturalistic data. For decades, processing acoustic stimuli has been a key training component of undergraduate and graduate education in language sciences. These trainings must continue and should not be replaced by computational toolkits. In the current climate, where AI and computational systems are considered as helpful “agents” for researchers, we caution the reader not to treat our toolkit as an agent. Research must be deployed, conducted, and interpreted by humans. Importantly, while fine-tuning and training our toolkit is possible, this requires substantial amount of energy use. Therefore, we caution our community to use these tools with a critical lens (Guest et al., 2025).

### Challenges in gathering naturalistic speech data

One major hurdle in collecting naturalistic speech is the classic observer’s paradox (Labov, 1972). In experimental settings, the very act of observation can alter how people speak. Speakers who are aware they are being recorded often adjust their speech, consciously or unconsciously, resulting in behavior that may not reflect their everyday speaking patterns. This paradox makes it extremely difficult to capture “completely naturalistic” speech in any research setting. Of course, such influences can be impacted by the task demands that participants complete or speakers’ awareness. Highly controlled laboratory tasks, while ensuring experimental precision, tend to produce speech that is more formal or hyper-articulated than normal conversation. Certain sociolinguistic variables may never emerge in a lab because participants modify their language when they know they are being observed. Consequently, spontaneous and naturalistic language samples are relatively rare in the literature despite their importance for revealing the diversity of language use in context.

Additional methodological challenges complicate the procurement of naturalistic speech data. Field recordings or storytelling/interview-style conversations are powerful ways to collect naturalistic data, but they also come with many challenges. They require extensive logistical preparation (i.e., site visits, recruitment) and attention to researchers’ positionality (i.e., is the researcher a community member?). For instance, understanding a speaker’s linguistic background or dialect usage is crucial for many studies, yet gathering this information accurately as a researcher can be complex. Speakers frequently navigate multiple dialects or languages in their lives, and traditional lab studies struggle to capture this linguistic diversity. This has been well documented in bilingualism studies, where it is challenging to capture language users’ fluid states of language experience (Surrain & Luk, 2019). Such background variation can directly affect the validity of controlled production data, since a “one-size-fits-all” lab task may not account for how diverse linguistic experiences shape speech perception and production. Moreover, structured experiments often target specific phenomena in isolation and may miss the broader spectrum of variability present in unmonitored conversation. For example, Bacon et al. (2019) found that naturalistic language samples from toddlers with autism revealed aspects of language ability that standard assessments overlooked (Bacon et al., 2019). Importantly, laboratory tasks like reading word lists or repeating sentences have inherent limitations. Reading aloud engages literacy skills and careful enunciation is not representative of everyday speech practices. These methods can complicate experimental designs where linguistically diverse individuals are tested. For instance, many heritage bilingual individuals are fluent in conversational skills, but they might not have reading skills in their heritage language due to inequalities in education practices and lack of access to heritage languages (Kircher & Kutlu, 2023; Kutlu & Pascual y Cabo, 2024). Recording heritage bilingual individuals using a reading task can become an immediate challenge as the task might not represent their conversational skills in their heritage language(s). Finally, due to funding limitations to language sciences, it is becoming more and more complicated and challenging to create open access speech corpora. Such limitations enforce inequalities, particularly for graduate students, early career scholars, and scholars who study minoritized language experiences.

### The need for naturalistic speech data

The scarcity of robust naturalistic corpora has direct implications for theory in linguistics and cognitive science (Kutlu & Hayes-Harb, 2023). Without ample datasets reflecting the true variability of speech, we risk building theoretical frameworks upon incomplete evidence (see critiques, Apfelbaum et al., 2022; Blasi et al., 2022; McMurray et al., 2023). Many foundational models in speech sciences as well as cognitive science implicitly assume relatively uniform data – often an idealized, noise-free representation of speech sounds. Such models can struggle to accommodate real-world linguistic diversity and may continue to misrepresent diversity as noise.

Recognizing this, many scholars advocate combining evidence from both controlled and spontaneous speech to gain a complete picture of language behavior. Controlled experiments remain invaluable for isolating specific variables and testing causal hypotheses. At the same time, spontaneous-speech data provide essential context and reveal emergent phenomena that structured tasks might never elicit. A balanced approach is needed in which laboratory findings are continually validated and supplemented with observations from natural communication. Indeed, multiple studies concur that neither approach alone is sufficient for understanding speech development and use. The challenge, however, has been practical: naturalistic data are difficult to collect and analyze without substantial research support mechanisms. Traditional sociophonetic fieldwork yields rich data but from relatively few speakers because recruiting participants, making recordings, and manually annotating speech require intensive effort. Even once audio is collected, transcribing and measuring phonetic details for hundreds of hours of spontaneous conversation is prohibitively time-consuming. It has been observed that day-long recordings used in child language studies can produce far more data than any single lab could manually annotate in a reasonable time (Casillas & Cristia, 2019; Räsänen et al., 2021). Therefore, automatic or semi-automatic tools have thus become essential for handling such corpora.

This is especially true as the field expands to include understudied languages and communities, where data may be accessible (through social media or community recordings) but access to research funds are scarce which limits the amount of time experts can be hired for. Leivada et al. (2019) enumerate numerous experimental challenges when attempting to elicit big data from speakers of less-common languages, highlighting that overcoming resource limitations is key to progress (Leivada et al., 2019). All these factors point to an urgent need for methodological innovation.

Fortunately, recent open-access computational tools offer a path forward. For decades, computational tools and models have been used in studying various aspects of cognition, including language, in tightly controlled ways. In the last several years, various toolkits were published that allow speech scientists to transcribe speech, extract acoustic features, and even diarize long conversations. These advances make it possible to contemplate fully automated pipelines for analyzing large volumes of conversational speech – something that was not feasible just a decade ago.

Importantly, we now live in a world where vast amounts of naturalistic speech are freely available online. Digital platforms (e.g., YouTube, Twitch) have created a rich set of recorded naturalistic speech data: everyday conversations on podcasts, unscripted discussions in online videos, interviews, dialogues in television and streaming media, and more. However, turning these rich resources into usable research data is non-trivial. As with other recording techniques (e.g., LENA recordings, LENA Research Foundation, 2011), processing even a fraction of this spontaneous speech without automated assistance would be impractical.

Here, we introduce a novel open-source toolkit for automatically obtaining naturalistic speech data from virtually any audio or video source (e.g., YouTube videos, podcasts, or recordings created by researchers) and converting it into an analyzable form. We integrate multiple different computational components for speaker diarization, speech transcription, and acoustic–phonetic analysis into a single toolkit, enabling researchers to easily perform fine-grained analyses (such as vowel formant extraction) on multi-speaker conversational data at scale. We demonstrate the toolkit’s utility with a case study on an American political debate, illustrating how large volumes of real-world speech can be collected and examined to address the need for naturalistic data in speech sciences. By automating what would otherwise be labor-intensive steps, the toolkit drastically lowers the barrier to using naturalistic speech in research. We introduce these processes step-by-step below. Our code is publicly available and can be found here: https://github.com/sarmadchandio/tapa.

## Methods

### Overview

TAPA consists of several interconnected components designed to facilitate comprehensive acoustic–phonetic analysis of naturalistic speech data as well as scripted speech samples. Each component addresses a specific challenge in processing and analyzing conversational audio. The first challenge is audio acquisition. While some scholars have funds to record speakers from various backgrounds, many scholars, particularly those who are in their early careers (e.g., graduate students, postdoctoral scholars, tenure-track or non-tenure-track faculty, or researchers who face various systematic challenges in different institutional or private areas), still struggle with audio acquisition. To address this challenge, we created a YouTube audio downloader to easily obtain any amount of naturalistic speech data from online sources, making it particularly useful for analyzing public speaking events, conversations, interviews, and debates that were posted on YouTube and subsequently converting them to audio files. While this solution does not solve the systematic issues that many scholars face, it allows scholars to work with naturalistic data without spending a significant amount of time solving download and conversion problems. The reader should note two important factors. First, TAPA also works with audio files outside of YouTube. For the Colab version, users upload the file to their Colab session storage or mount it from Google Drive; for the local console version, users provide a path to the audio file on their machine. Both options are presented on our GitHub page. Second, downloading content from YouTube may be restricted depending on the researcher’s location or on YouTube’s terms of service. TAPA itself uses only open-access toolkits, but private companies frequently change their regulations, and tools that are currently open-access may become inaccessible in the future. While we cannot predict such changes, our GitHub page will let us maintain version control and share updates with the community. Additionally, we included browser cookies as a default option to ensure that working with YouTube videos remains a stable feature.

**Fig. 1:**
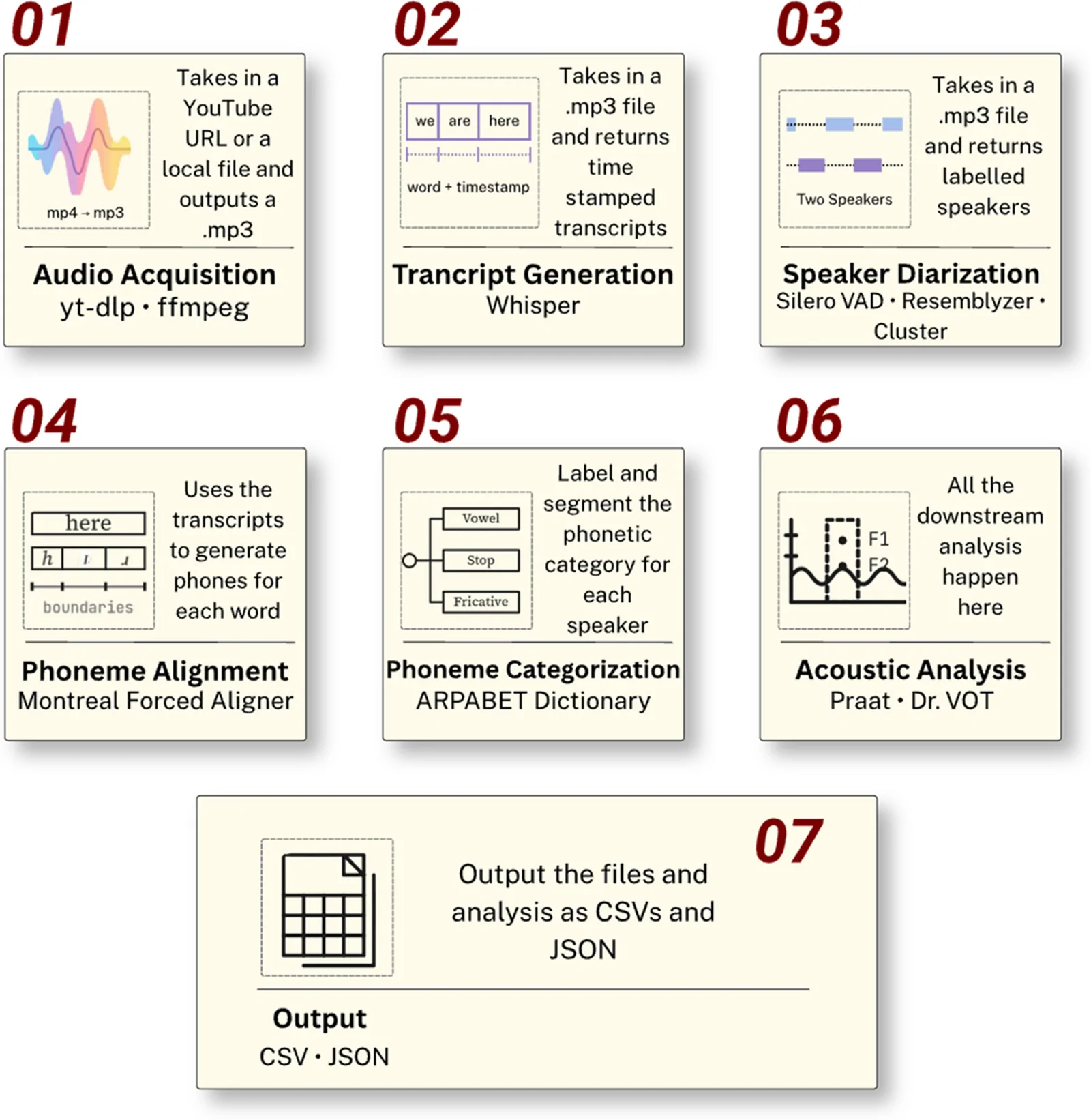
The pipeline’s components are shown in boxes. The process starts with “YouTube audio downloader” and ends with the “Output” node

The second part of TAPA innovation is that it goes through transcription uses and speaker diarization which is a component that identifies and separates different speakers within the audio, enabling speaker-specific analysis in multi-participant conversations. Then, we transcribe the entire audio file and use other components to prepare for the phonetic analysis, which is described in detail below. Figure 1 visually presents each component and step.

These components can be used independently or combined into a complete pipeline (as shown in Fig. 1) for analyzing conversational speech data, from acquisition through acoustic–phonetic analysis.

### Requirements

#### Core dependencies

Yt-dlp (Yt-Dlp, n.d.) is a library that facilitates accessing and downloading YouTube data via YouTube URLs. We utilize its features in the YouTube Audio Downloader module of the pipeline to extract each video’s audio stream and convert it to an.mp3 file.

PyTorch (Paszke et al., 2019) is a deep-learning framework that powers the neural-network components of TAPA’s speech-processing pipeline. Transcription is performed by OpenAI’s Whisper (Radford et al., 2023), which produces word-level transcripts with timestamps. Speaker diarization is built from two pretrained models loaded via PyTorch. Silero VAD detects speech regions from the audio (separating human speech from silence, noise, and other sounds), and Resemblyzer maps each detected region to a 256-dimensional voice embedding in which clips from the same speaker sit close together and clips from different speakers sit far apart (Silero Team, 2024). These embeddings are then clustered to associate speech with different speakers.

Montreal Forced Aligner (MFA) (McAuliffe et al., 2017) is an open-source model that has been actively used for many years by speech scientists. We incorporated MFA to produce precise phone-level boundaries from a paired audio file and transcript.

Librosa (McFee et al., 2023) is a Python package designed to handle audio file processing and feature extraction. Its main role in the pipeline is to sample the audio data files to retrieve information during the phoneme analysis stage. Praat-parselmouth is a port of the Praat software for Python (Jadoul et al., 2018). Praat software is a common open-source software used by linguists to measure and analyze recorded speech and its features. Thus, we integrate Parselmouth into the toolkit during the formant analysis phase to extract F1 and F2 values for vowels. Additionally, we use Dr. VOT, which is another open-source component (Shrem et al., 2019). It uses a deep-learning model that predicts Voice Onset Time directly from the speech waveform. We used Dr. VOT for the stop consonant analysis. Unlike Parselmouth’s signal-based heuristic, Dr.VOT produces explicit burst and voicing-onset boundaries and handles both positive (aspirated) and negative (prevoiced) VOT. We retain the Parselmouth measurement as a per-token fallback for stops that Dr. VOT cannot process.

#### System requirements

We recommend a Python virtual environment to isolate the dependencies mentioned above. The development and testing of the pipeline were performed on an Ubuntu 22.04.3 LTS operating system. We also validated the implementation of the pipeline via the NVIDIA T4 GPU on Google Colab (using a Windows and a Mac computer) to facilitate PyTorch and Transformer operations.

#### Installation process

Detailed installation and usage instructions are available at: https://github.com/sarmadchandio/tapa. While GPU acceleration (use of a Graphics Processing Unit, providing high-volume and high-speed parallel processing of data) via PyTorch is recommended for optimal performance, the toolkit will function on CPU-only systems (systems with a Central Processing Unit, the primary processing unit of any computer) with reduced processing speed. 

#### Cloud-based installation

The toolkit can also be used more accessibly in Google Colab. This provides users immediate access to GPU resources without local setup. The GitHub page provides Google Colab setup. We recommend users with limited coding background to use the cloud-based installation option. Users who actively use Python-based code can copy our code and make adjustments or customizations to it. Please note that while TAPA itself does not transmit or store any user data, audio files processed through the Google Colab version are uploaded to Google’s servers for the duration of the session, and Google’s own data-handling policies apply. Users working with audio that is not publicly available, or that contains identifiable speakers, should consult their institutional review board about appropriate data-handling procedures before uploading such files to Colab. The local installation of TAPA runs entirely on the user’s own machine and does not send audio to any external server.

### Components

#### Audio acquisition

The YouTube Audio Downloader module accepts any standard YouTube URL (youtube.com/watch?v = …, youtu.be/…,/shorts/…,/embed/…) and downloads the audio of the video as an.mp3 file. Its main dependency is yt-dlp (Yt-Dlp, n.d.), an actively maintained media extractor that retrieves a video’s best available audio stream from YouTube’s public endpoints. The output.mp3 files are named after each video’s 11-character YouTube identifier (the v = … string), so all downstream pipeline outputs (e.g., < id > _diarization.csv) trace directly back to their source recording. As mentioned above, due to YouTube’s regulations, some users might not be able to download YouTube videos as audio files. In those cases, we recommend that users find alternative ways to record the audio from YouTube. Based on the user’s preference, once an audio file is available, they can freely use the rest of TAPA components.

#### Transcript generation

The transcription module takes an.mp3 file as input and produces a word-level transcript with timestamps. It uses OpenAI’s Whisper (default small.en for English). Users can change the Whisper model from small.en to larger models or they can use multilingual models. Whisper produces word-level boundaries, so each transcribed token carries start and end times in seconds. The output is a CSV file of (word, start, end, speaker) tuples that downstream stages use both for forced alignment and for associating words with diarized speakers. Processing is automatically accelerated using an NVIDIA graphics card (GPU) when one is available, which can be significantly faster than running on the CPU alone. However, it falls back to CPU automatically if no compatible GPU is found.

#### Speaker diarization

The Speaker Diarization module takes an.mp3 file input and identifies who is speaking and when. It is built around two pretrained components: Silero VAD, a lightweight neural voice-activity detector that returns speech-active segments, and Resemblyzer, a generalized end-to-end speaker-verification encoder that produces a fixed-length embedding per voice (Silero Team, 2024). VAD-detected segments are embedded with Resemblyzer and then clustered into speaker groups; the number of speakers can be either supplied by the user or auto-detected. The output is a CSV of speaker-labeled utterance timestamps. The same module is responsible for assigning each transcribed word to the speaker whose segment overlaps its midpoint.

#### Phoneme alignment

Phoneme boundaries are obtained from the Montreal Forced Aligner (McAuliffe et al., 2017) (using its English-US ARPA acoustic and pronunciation models) when it’s available, otherwise we use a CMUdict-based (Weide, 1998) proportional-timing fallback to ensure that the pipeline is not guessing where the sounds occur. At this stage, sounds that are identified are categorized into vowels, stops, and fricatives using ARPAbet-to-IPA mappings and per-class duration filters.

#### Vowel formants

Vowel formants are measured with Praat-parselmouth (Jadoul et al., 2018), a Python port of the Praat phonetics software. F1 and F2 are measured as the mean across the trimmed interval using the Burg algorithm. The outer 15% on each side of the vowel interval is trimmed before measurement to suppress coarticulatory transitions. The maximum-formant ceiling is set adaptively from each token’s median F0: 5000 Hz when median F0 is below 170 Hz and 5500 Hz at or above 170 Hz, which applies a lower ceiling to typically lower-pitched voices and a higher ceiling to typically higher-pitched voices. Median F0 is estimated over the same interval (pitch floor 75 Hz, ceiling 600 Hz). Tokens whose measured formants fall outside plausible bounds, or for which F1 ≥ F2, are discarded.

#### Stop consonants VOTs

Stop Voice Onset Time is measured with Dr.VOT (Shrem et al., 2019), a neural model that predicts burst and voicing-onset boundaries directly from the waveform and natively handles both positive (aspirated) and negative (prevoiced) VOT. Tokens that Dr.VOT cannot score fall back to a Parselmouth-based signal heuristic, so coverage never drops below the Praat baseline.

#### Fricative spectral moments

The four spectral moments, which are center of gravity, spectral standard deviation, skewness, and kurtosis (Forrest et al., 1988; Jongman et al., 2000), are computed via Praat-Parselmouth’s (Jadoul et al., 2018) spectrum object in the middle 80% of each fricative to suppress vowel transitions. Tokens with an implausible center of gravity (outside 0.5–12 kHz) are discarded.

Once the phonetic analyses are conducted, TAPA creates the output documents, which are available as a.csv or.json file. These files contain per-token acoustic measurements and per-speaker, per-sound summary statistics for all three segment classes.

### Case study: Presidential debate

We used the toolkit on the 2016 U.S. presidential debate between Hillary Clinton and Donald Trump (Television, 2016). The recording is approximately 90 min of moderated multi-speaker speech with audience presence, podium reverberation, occasional overlapping talkers, and the prosodic and articulatory adjustments candidates make in performative political contexts. We chose this recording to illustrate the range of naturalistic audio conditions the toolkit is intended to handle outside the laboratory. Across the full debate, the pipeline extracted 19,855 vowel tokens, 6547 stop tokens, and 6372 fricative tokens. The analyses below focus on the two main speakers, Donald Trump and Hillary Clinton (DT and HC henceforth), with the remaining tokens distributed across moderators and audience-question readers. The per-speaker counts that the case-study analyses draw on are as follows: 8395 vowel tokens for DT and 8675 for HC across all categories, of which 7124 (DT) and 7475 (HC) fall into the ten monophthong categories used for vowel-space analyses; 2749 stop tokens for DT and 2893 for HC; and 2691 fricative tokens for DT and 2794 for HC.

The debate audio is professionally recorded, which mitigates some acoustic challenges such as low SNR and microphone noise that would arise with lower-quality field recordings. It nonetheless retains the features that distinguish naturalistic from laboratory speech listed above. We trained the acoustic components in our pipeline on diverse datasets and expect them to generalize across recording conditions, but this case study cannot establish the full range of conditions under which the toolkit performs reliably. Future work will systematically evaluate performance across recording qualities, speaker populations, and speech styles.

## Results

### Validation

To enable validation against expert annotation, a single phonetically trained RA produced all hand-coded measurements from a stratified sample of tokens from the 2016 debate audio (Television, 2016) without access to the pipeline’s output. Inter-rater reliability between human coders was therefore not assessed here; the validation is instead a comparison between automated pipeline output and a single expert annotator.

For vowels, 240 monophthong tokens were drawn across the 90-min debate (60 per speaker per debate phase, with phases corresponding to the first and second halves of each speaker’s contributions), with F1 and F2 measured at the temporal midpoint of each vowel in Praat.

For stops, 120 tokens across the six American English stops (/p b t d k g/; 60 per speaker, split equally across phases) were identified and VOT measured as the interval between burst release and voicing onset. Each pipeline-extracted token was matched to the nearest hand-coded token by speaker, phonetic category, and time (3-s window for vowels, 1-s window for stops). Match rates were 240/240 (100%) for vowels and 112/120 (93%) for stops. The eight unmatched stops were tokens for which forced alignment did not place a stop within the search window of the RA-coded time.

For fricatives, we drew a separate stratified random sample of 160 voiceless tokens (20 per speaker per phone across/s/,/ʃ/,/f/,/θ/) from the pipeline’s MFA-aligned fricative intervals. Each token was extracted from the original audio file using FFmpeg (FFmpeg Developers, 2024) into an individual 16-bit, mono, 44.1-kHz WAV file with a 5-ms pad on each side, paired with a programmatically generated Praat TextGrid carrying the fricative interval labeled in IPA. Hand-coded spectral moments (center of gravity, spectral standard deviation, skewness, and kurtosis) were computed using a time-averaged-spectrum (TAS) procedure (DiCanio, 2013), in which the magnitude spectrum is calculated from the average of multiple overlapping discrete Fourier transforms across the center of the fricative interval. Specifically, we used DiCanio’s (2013) TAS script(DiCanio, 2013), which implements the moment-calculation procedure proposed by Forrest and colleagues (Forrest et al., 1988). Tokens shorter than 56 ms were excluded because the TAS procedure requires sufficient duration for six overlapping 15-ms analysis windows. This exclusion removed 11 tokens, leaving 149 in the final hand-coded sample. The resulting log files were merged with the pipeline’s per-token output by clip filename to yield 149 paired hand-coded and pipeline measurements. Across all three categories, agreement was evaluated with Pearson correlation, mean absolute error, and paired-difference bias using RStudio (version 4.3.1) (Posit team, 2026). All visualization was done by using ggplot2 library in R (Wickham, 2016).

The validation reported here establishes pipeline accuracy on a substantial sample of debate audio but is necessarily scoped to what the present study could examine. All measurements come from two speakers of American English in a single recording context, and other components such as diphthongs, voiced stops, and suprasegmental features (e.g., prosody) were not evaluated. The VOT results identified a training-deployment mismatch in the Dr.VOT backend that we are actively addressing through alternative algorithms. Extension to other languages, speech styles, and recording conditions is a priority for future work, since the pretrained components in the pipeline may behave differently outside the conditions in which they were trained. We view the present validation as sufficient to support the case-study claims about vowel space, voiceless stop consonants, and fricative-spectrum characterization in this dataset, with broader validation across languages, registers, and acoustic conditions left for subsequent studies.

### Vowel formant validation and observations

Of the 240 hand-coded vowel tokens, the pipeline produced a same-category match within 3 s in 226 cases (94.2%). The 14 unmatched tokens were RA-coded vowels for which the pipeline did not place the same vowel category within the search window; these were excluded from formant agreement statistics. Unmatched tokens were distributed across the high and front categories (/u/,/æ/,/i/) where forced alignment occasionally placed the pipeline’s nearest same-category token outside the 3-s window. For the 226 matched tokens, agreement between pipeline-extracted formants and human Praat measurements was high. For F1, the Pearson correlation was *r* = 0.886 (95% CI [0.854, 0.911]), mean absolute error (MAE) = 37 Hz, and a non-significant bias of + 6 ± 67 Hz (*t*(225) = 1.40, *p* = 0.164). For F2, agreement was *r* = 0.865 [0.828, 0.894], MAE = 125 Hz, with a small but reliable negative bias of − 40 ± 181 Hz (*t*(225) =  − 3.31, *p* = 0.001). Per-vowel mean absolute errors ranged from 10 to 70 Hz for F1 and from 51 to 217 Hz for F2 across categories and speakers (see Table 1), with the largest errors in/u/and/ɪ/(back and high vowels for which formant estimation is more sensitive to small windowing differences).

**Table 1:** Per-vowel, per-speaker MAE and bias for F1 and F2 in the 224 matched validation tokens

Speaker	Vowel	*n*	F1 MAE (Hz)	F2 MAE (Hz)	F1 bias	F2 bias
DT	i	20	27	102	+ 4	− 43
DT	u	19	28	217	+ 9	− 191
DT	æ	20	53	117	− 16	− 51
DT	ɛ	19	38	139	+ 23	− 67
DT	ɪ	20	50	210	− 22	− 51
DT	ʊ	20	49	117	− 14	− 84
HC	i	18	19	82	+ 5	+ 47
HC	u	15	10	77	− 1	− 42
HC	æ	17	44	51	+ 23	+ 23
HC	ɛ	19	70	139	+ 54	− 10
HC	ɪ	20	32	113	+ 5	+ 97
HC	ʊ	19	19	111	+ 10	− 100

Bias = pipeline minus human; negative values indicate the pipeline measured lower than the RA. DT stands for Donald Trump, and HC stands for Hillary Clinton

For applications focused on vowel space characterization, sociophonetic variation, or longitudinal speaker tracking, the pipeline’s accuracy is sufficient to aid researchers in their annotation, with the caveat that researchers using the toolkit on noisier audio or on phenomena that depend on fine F2 resolution (e.g., subtle dialect differences in/æ/raising or/u/fronting) should validate against a hand-coded subsample of their own corpus.

**Fig. 2:**
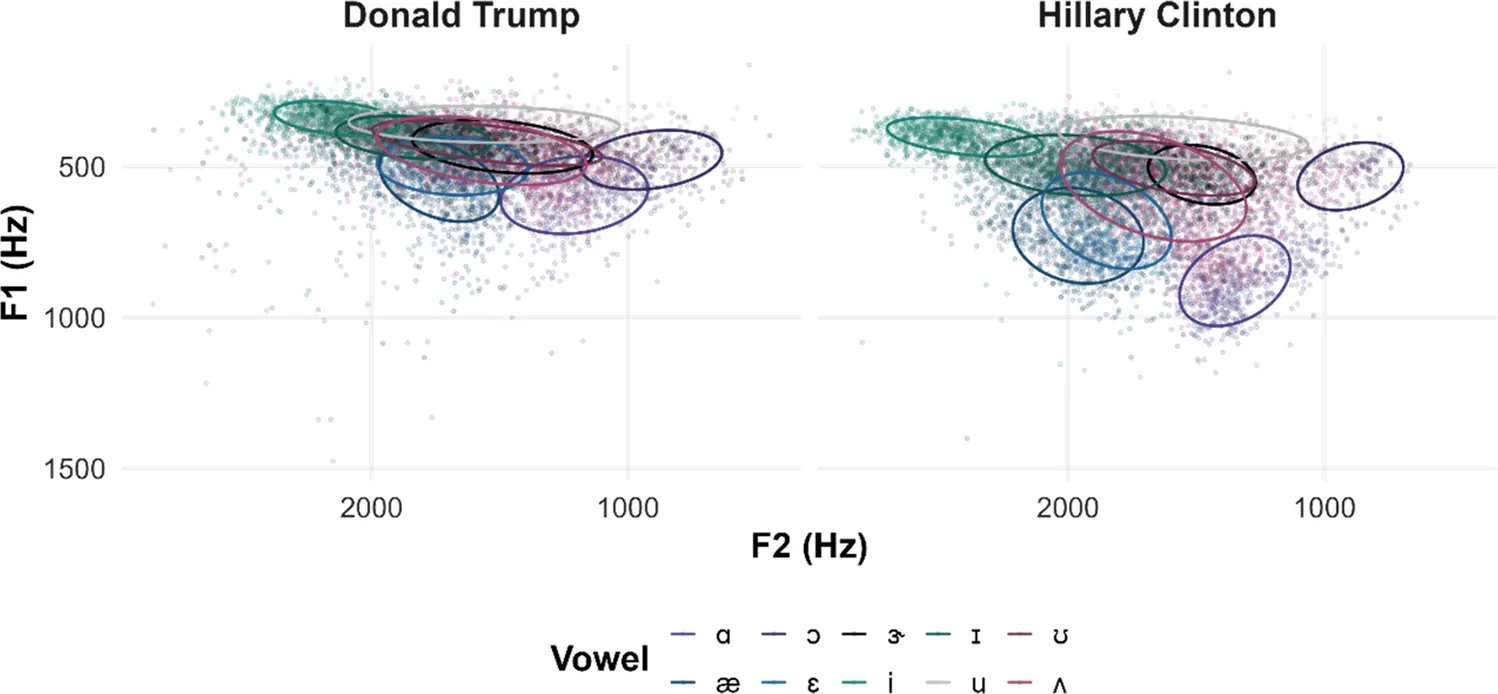
F1 × F2 vowel spaces for Donald Trump (*left*) and Hillary Clinton (*right*) in the 2016 U.S. presidential debate. *Scatter points* show individual vowel tokens extracted by the pipeline; *ellipses* show  confidence contours for each monophthong category. Axes are inverted in the traditional phonetic convention (F2 decreasing rightward, F1 decreasing upward)

Figure 2 shows the F1 × F2 vowel spaces of the two speakers. Two features are immediately visible. First, Clinton’s vowel categories have higher F1 and higher F2 values in Hz than Trump’s, which places them toward the lower-left of the plot given the inverted phonetic-convention axes. Second, Clinton’s vowel categories appear to occupy a larger total area, with the corner vowels/i/,/æ/,/ɑ/, and/u/pushed further outward than Trump’s.

To anchor the case-study measurements against published norms, we overlaid the Hillenbrand and colleagues’(Hillenbrand et al., 1995) means for adult American English men and women onto the same F1 × F2 dimensions. This dataset comprises 45 men, 48 women, and 46 children producing 12 English monophthongs in/hVd/syllables. Trump’s category means deviated from the Hillenbrand-men reference by F1 MAE = 72 Hz and F2 MAE = 230 Hz across the ten shared categories; Clinton’s deviated from the Hillenbrand-women reference by F1 MAE = 68 Hz and F2 MAE = 287 Hz (Table 2). Both speakers’ F1 values were below the Hillenbrand reference (F1 bias =  − 71 Hz for Trump, − 53 Hz for Clinton), consistent with the well-documented tendency for vowels in continuous speech to be more centralized than vowels produced in isolated/hVd/words (Lindblom, 1963; Strange & Jenkins, 2013). For F2, Trump’s vowels showed a small backward bias (F2 =  + 68 Hz higher than reference) and Clinton’s a forward bias (F2 =  − 95 Hz lower).

**Table 2:** Mean deviation of debate speakers’ vowel category means from same-sex Hillenbrand et al. (1995) reference values, computed across the ten shared monophthong categories

Comparison	Vowels (*n*)	F1 MAE (Hz)	F2 MAE (Hz)	F1 bias	F2 bias
Trump vs. Hillenbrand men	10	72	230	− 71	+ 68
Clinton vs. Hillenbrand women	10	68	287	− 53	− 95

Bias = debate minus reference

### Stop and fricative validations and observations

For the present case study, we used the Dr.VOT because it has been previously validated against expert annotation (Shrem et al., 2019). We restricted the case-study analysis to voiceless stops (/p t k/), for which English phonology licenses a positive-VOT sign. The mean unsigned VOT magnitudes fell in the expected American English long-lag range across both speakers:/p/~ 72 ms,/t/~ 69 ms, and/k/~ 83 ms (see Table 3 below), values consistent with published norms for American English voiceless onset stops in connected speech (Lisker, 1986). However, two important limitations emerged. Dr.VOT was trained on four corpora of isolated single-word productions, with stop classes rebalanced during training to a 50:50 positive-to-negative ratio (the natural ratio in the underlying corpora is 80:20). On the debate audio, this training-deployment mismatch surfaced as a tagging problem: Dr.VOT labeled a large share of voiceless onset stops as prevoiced (NEG_VOT), at rates of 45–61% across/p t k/for both speakers (Table 3). This is incompatible with the phonology of American English, where voiceless onset stops are positive-VOT in essentially all tokens. The misclassification reflects the combined effect of the class-balanced training prior and the shift from isolated single words to continuous, noisy debate audio.

**Table 3:** Per-speaker voiceless stop VOT from the Dr.VOT backend on the 2016 debate

Speaker	Stop	*n*	VOT mean (ms)	VOT SD (ms)	NEG_VOT rate
DT	/p/	317	77.1	80.3	54.6%
DT	/t/	881	70.9	70.4	51.6%
DT	/k/	432	82.7	75.3	60.9%
HC	/p/	333	74.0	74.0	47.4%
HC	/t/	1022	65.9	69.2	50.0%
HC	/k/	445	81.0	76.1	59.3%

VOT is the unsigned mean of the burst-to-voicing magnitude. NEG_VOT rate is the share of tokens Dr. VOT’s classifier labeled as prevoiced

Validation against the RA-coded VOT measurements confirmed a second issue: the per-token magnitudes Dr.VOT reports do not consistently match expert measurements on the same tokens. Restricting to the 55 matched voiceless stops (where the RAs’ positive durations and Dr. VOT’s VOT magnitudes should align if the segmentation head is working), agreement was poor (*r* =  − 0.04 [− 0.30, + 0.23], MAE = 75.8 ms, bias =  + 35.6 ± 85.9 ms; *t*(54) = 3.07,* p* = 0.003). In other words, the aggregate long-lag means reported above look phonetically reasonable, but Dr. VOT’s per-token boundary placement on continuous, noisy speech diverges from where a human coder would place the same boundaries by approximately 50–80 ms on average.

We therefore restrict our case-study consonant analyses to voiceless onset stops and report unsigned VOT magnitudes only, treating the per-category means as descriptive aggregates rather than per-token measurements. For voiced stops, the picture is more difficult: Dr. VOT’s magnitudes for/b d g/mix two physically different intervals (burst-to-voicing for tokens labeled POS_VOT vs. voicing-lead-to-burst for tokens labeled NEG_VOT) depending on the sign decision, so a single VOT number for voiced stops is not directly interpretable without per-token verification. For this reason, we omit voiced stops from the case-study table. However, additional information about the voiced consonants can be found in our OSF page.

In terms of fricative consonants, fricative spectral moments showed the expected/s/–/ʃ/contrast for both speakers, with/s/exhibiting the highest center of gravity (CoG) (Trump 4369 Hz; Clinton 5279 Hz) and/ʃ/a substantially lower CoG (Trump 3292 Hz; Clinton 3560 Hz). The non-sibilants/f/and/θ/fell below the sibilants overall, though their CoG values overlapped with/ʃ/for Trump (/f/3468 Hz;/θ/2151 Hz) and were more clearly separated for Clinton (/f/3021 Hz;/θ/1952 Hz). Clinton’s/s/CoG was approximately 910 Hz higher than Trump’s, consistent with the tendency for feminine-presenting speakers to produce/s/with higher spectral mean than masculine-presenting speakers (Stuart-Smith, 2020).

We validated all four spectral moments against 149 hand-coded measurements computed with a time-averaged-spectrum (TAS) procedure (see Methods). Across moments, the pipeline showed the strongest agreement on spectral standard deviation (*r* = 0.80, MAE = 446 Hz), followed by CoG (*r* = 0.69, MAE = 1118 Hz), kurtosis (*r* = 0.62, MAE = 2.2), and skewness (*r* = 0.60, MAE = 0.9; see Table 4). The pipeline systematically under-estimated CoG by 1108 Hz (*t*(148) =  − 12.34, *p* <.001) relative to hand coding, a bias consistent with the difference in low-frequency preprocessing between the two methods: the hand-coded TAS applies a 300 Hz high-pass filter and time-averages six 15 ms windows across the central 80% of the fricative, whereas the pipeline computes a single Fourier transform over the entire fricative interval without low-frequency filtering. This methodological difference might disproportionately lower pipeline CoG when low-frequency energy from room reverb and audience noise is included in the calculation.

**Table 4:** Pipeline vs. hand-coded agreement across all four spectral moments

Moment	*n*	*r* [95% CI]	MAE	Bias
Spectral SD (Hz)	149	0.80 [0.74, 0.85]	446	− 354
CoG (Hz)	149	0.69 [0.59, 0.76]	1118	− 1108
Kurtosis	149	0.62 [0.51, 0.71]	2.2	− 0.6
Skewness	149	0.60 [0.49, 0.69]	0.9	− 0.4

Bias = pipeline minus hand-coded; negative values indicate the pipeline measured lower than the hand-coded reference

Per-phone agreement on CoG followed the well-documented sibilant/non-sibilant divide. Pipeline–hand-coded agreement was strong for the sibilants (/ʃ/*r* =.94, *n* = 37, MAE = 221 Hz;/s/*r* =.87, *n* = 38, MAE = 557 Hz) and substantially weaker for the non-sibilants (/f/*r* =.46, *n* = 38, MAE = 2037 Hz;/θ/*r* =.59, *n* = 36, MAE = 1664 Hz). The qualitative pattern/s/>/ʃ/>/f/,/θ/and the higher CoG for Clinton’s/s/relative to Trump’s were preserved in both measurement procedures. The poorer non-sibilant agreement reflects the intrinsic acoustic properties of/f/and/θ/rather than a pipeline-specific limitation: non-sibilant fricatives are spectrally indistinct from one another(Jongman et al., 2000), and lab-clean discriminant classification of/f/vs./θ/from moments alone has historically been near chance.(Forrest et al., 1988) For substantive case-study claims, we therefore consider the pipeline’s sibilant CoG measurements quantitatively trustworthy and treat its non-sibilant CoG, skewness, and kurtosis values as descriptive aggregates rather than per-token measurements. Improving non-sibilant analysis through time-averaged-spectrum integration and 300-Hz high-pass filtering is a priority for future versions of the toolkit.

## Discussion

The goal of this project was to create open-access tools for researchers who are interested in collecting and analyzing naturalistic speech samples. The Toolkit for Acoustic–Phonetic Analysis (TAPA) integrates open-source components into a single Python-based pipeline that can be installed with one command and run from a Google Colab notebook with no local setup. We used this toolkit on the 2016 U.S. presidential debate, the toolkit extracted nearly 33,000 vowel, stop, and fricative tokens from a single 90-min recording and produced measurements that agree with phonetically trained hand annotation to within the range typical for expert hand-coding of continuous speech. We treat the case study as a demonstration of what the pipeline makes available, and the validation against hand annotation as a demonstration of where its outputs can be trusted and where they cannot.

### Naturalistic data and the role of automated pipelines

Naturalistic speech data are essential for advancing theory in the speech sciences. As we argued in the introduction, many of the dominant models of speech perception and production were built on data drawn primarily from laboratory tasks involving read or repeated speech, and the resulting frameworks struggle to accommodate the variability that characterizes real-world language use. Field recordings, longitudinal child-centered recordings, and conversational corpora have all made important contributions, but each comes with substantial logistical and annotation costs that limit who can produce them and at what scale. The open-access tools produced in speech technology such as automatic speech recognition, forced alignment, speaker diarization, and acoustic feature extraction makes it feasible to process volumes of conversational audio that would have required years of manual annotation a decade ago. The contribution of TAPA is to package these components together in a way that lets researchers move from a YouTube URL or a local audio file to per-speaker, per-phone acoustic measurements without writing custom code, while preserving the ability to inspect, swap, or override any component in the chain.

The pipeline complements rather than replaces existing tools such as LENA (LENA Research Foundation, 2011) and ALICE (Räsänen et al., 2021). These tools can operate at the level of vocalization counts, turn counts, and syllable counts, and are designed primarily for child-centered daylong recordings with a single wearable microphone. TAPA operates at the level of individual phones (i.e., vowel formants, stop VOT, fricative spectral moments) and is designed for multi-speaker audio from any source. The Montreal Forced Aligner (McAuliffe et al., 2017), one of the components TAPA integrates, is a specialized tool for producing phone alignments from audio and a transcript; TAPA’s role is not to replace MFA but to wrap the larger workflow around it, from audio acquisition through diarization, transcription, and per-segment acoustic analysis. The same modular logic applies to the other components: users can substitute each with an alternative of their choosing. In addition, the users can change the settings based on their needs. For instance, larger multilingual models can be used instead of the current small English model that was used in this case.

### The pipeline is a tool and should not replace trained phoneticians

The validation against hand annotation produced two qualitatively different results that, in our view, define the more important contribution of this work. For vowel formants, agreement with the RA was high, within the range expected for expert hand-coding of continuous speech. However, it should be noted that researchers working on noisier audio or on phenomena requiring fine F2 resolution should validate against a hand-coded subsample of their own corpus.

For stop VOTs, the picture is more nuanced. Dr.VOT decomposes each measurement into two parts: a segmentation step that locates the burst and voicing onset on the waveform, and a classifier that decides whether voicing follows the burst (positive VOT) or precedes it (negative VOT). On the case-study audio, the aggregate magnitudes for voiceless onset stops were in the expected American English long-lag range (means across speakers), suggesting the segmentation step produces roughly the right category averages on this subset. However, two problems emerged when we looked closer. First, Dr. VOT’s sign classifier labeled 45–61% of voiceless onset stops as prevoiced. This is a rate that is incompatible with the phonology of American English, where/p t k/in onset position are positive-VOT in essentially all tokens. Second, when we compared per-token magnitudes against the RAs’ measurements (restricting to the matched voiceless tokens), agreement was poor, indicating that the boundaries Dr.VOT places on continuous, noisy speech do not consistently match where a human coder would place them, even when the per-category averages come out plausible. We further chose not to report voiced-stop VOT at all in the case-study analyses, because Dr. VOT’s reported magnitude for/b d g/depends on the (unreliable) sign decision: a NEG_VOT-labeled/b/contributes its prevoicing-lead duration, while a POS_VOT-labeled/b/contributes its burst-to-voicing duration, and averaging across both without sign correction yields a number that is not interpretable as a single physical interval.

Both problems trace to the gap between Dr. VOT’s training data and our deployment conditions. The model was trained on four corpora of isolated single-word productions in clean recording environments, with stop classes rebalanced to a 50:50 positive-to-negative ratio (the natural ratio in the underlying corpora is 80:20 (Shrem et al., 2019)). Debate audio challenges both assumptions: it is continuous speech with audience noise, reverberation, and overlapping talkers, and the true positive-to-negative ratio for English voiceless onset stops is closer to 95:5 than to 50:50. While this is a limitation at the moment, it can be ameliorated with future training projects which TAPA will continue to work on.

The fricative validation surfaces a more nuanced picture than the vowel or stop analyses. Pipeline–hand-coded agreement varied systematically by both moment and place of articulation. Spectral standard deviation showed the strongest agreement, center of gravity was intermediate, and the higher-order moments skewness and kurtosis showed more modest agreement. Within center of gravity, sibilants tracked the hand-coded reference closely while non-sibilants did not. This place-of-articulation pattern, in which sibilants are characterized far more reliably than non-sibilants, has been previously documented in the fricative literature (Forrest et al., 1988; Jongman et al., 2000).

Three aspects of this pattern warrant closer interpretation. First, the more modest agreement for skewness and kurtosis is expected on statistical grounds. As higher-order moments, they describe how a spectrum departs from a Gaussian shape and are disproportionately sensitive to a small number of frequency bins at the spectral extremes. In naturalistic audio, a brief burst of audience noise, a trace of voicing in the analysis window, or a small segmentation error can shift these moments substantially while leaving center of gravity and spectral spread comparatively stable. Second, the pipeline’s systematic under-estimate of center of gravity is methodological rather than a flaw in the implementation. The hand-coded reference applies a low-frequency filter and averages several short windows across the center of the fricative, whereas the pipeline computes a single transform over the whole interval without low-frequency filtering. The retained low-frequency energy pulls the pipeline’s measurement downward, and because non-sibilant fricatives carry little high-frequency energy to begin with, that contribution matters more for them than for sibilants, which is why the offset is largest exactly where agreement is weakest. The two procedures measure the same construct through different preprocessing choices, and the offset between them is predictable from that difference. Third, these results point out to an important reporting strategy for researchers applying TAPA to their own fricative data. Sibilant center of gravity and spectral standard deviation can be reported as quantitative measurements, provided that inference rests on within-study contrasts rather than cross-study absolute values. Non-sibilant moments and the higher-order moments should be treated as descriptive aggregates accompanied by a hand-coded validation subsample from the target corpus. More broadly, the fricative findings illustrate the value of validating each measurement type against an independent gold standard before drawing substantive phonetic conclusions, particularly when an automated pipeline built for general phonetic analysis is applied to specialized segment classes whose acoustic properties demand tailored measurement procedures. Integrating a time-averaged-spectrum option with low-frequency filtering into the pipeline would narrow the gap across moments and is a priority for the next release.

### Limitations and future directions

TAPA inherits the limitations of the pretrained components it integrates. Whisper’s small.en model is English-only, MFA’s english_us_arpa acoustic model is American English, and Dr.VOT was trained on four English single-word corpora. The pipeline’s default configuration therefore reflects an American English-centric bias, and deployment on other varieties (e.g., different dialects, accents, registers) requires either swapping in language- or variety-appropriate models (Whisper has multilingual checkpoints; MFA has acoustic models for dozens of languages) or validating that the American English-trained defaults produce acceptable output on the target variety. The modular structure of the pipeline supports this kind of substitution by design, but we emphasize that successful substitution still depends on the user understanding the linguistic structure of their target variety and being able to evaluate the substituted model’s output against that knowledge. We see expanding validated coverage of additional varieties, particularly varieties used by communities historically underrepresented in speech-science training corpora, as an important direction for the community working with this kind of tool.

The Dr.VOT classifier discussion above is one instance of a more general issue: pretrained neural components are opaque relative to their training data and training procedure, and their performance on out-of-domain audio is hard to predict from the model alone. Although the TAPA pipeline is open-source and inspectable, the models inside it are pretrained on corpora and procedures that the average user cannot fully audit. We therefore recommend that any substantive deployment of TAPA include validation against hand-annotated data from the target corpus, and that publications using the toolkit report which component produced each measurement (segmentation versus classification heads in Dr.VOT, MFA versus the CMUdict fallback for phone alignment) so that downstream readers can evaluate which parts of the analysis depend on which model assumptions. The pipeline writes this information to its output files automatically, making it explicit in publications closes the reproducibility loop.

Other limitations bound the current implementation. Diarization handles non-overlapping multi-speaker audio reliably but degrades on heavily overlapping speech, a known limitation of clustering-based approaches (Park et al., 2022); the toolkit does not currently include a dedicated overlap-resolution stage. Forced alignment depends on the words appearing in the pronunciation dictionary, so some proper names and out-of-vocabulary tokens may be misaligned or skipped; the proportional-timing fallback when MFA is not installed produces usable but less precise boundaries. The current acoustic measurements cover vowels, stops, and fricatives but not flaps, taps, rhotics, nasals, or prosodic features such as intonation contours, speech rate, and rhythm; these are natural extensions that the pipeline architecture supports and that we view as future-work targets. Recording conditions matter: the case-study debate audio is moderately noisy by laboratory standards (audience presence, podium reverberation, occasional overlapping talkers), but the microphone quality may be better than some of the historical archival audio or field recordings. More scientific research is needed to evaluate different audio settings objectively. We note that building, validating, and documenting an integrated open-source pipeline of this scope is itself a substantial engineering and methodological effort. The current release represents the components we were able to validate at this stage and is intended as a working baseline rather than a finished product. Subsequent releases will incorporate the time-averaged-spectrum fricative procedure, an alternative VOT backend, and additional segment classes as validation against hand-coded data becomes available.

Crucially, we emphasize that TAPA is not a replacement for trained phonetic researchers nor phonetic training. It is research infrastructure that produces large-scale measurements from naturalistic audio, and the validation we have reported here defines the boundaries within which those measurements can be trusted. We strongly recommend that users perform small-scale, hand-coded verifications to confirm their results. Ultimately, by embracing naturalistic data through methods enabled by this toolkit, speech-science research can ensure that its theories of language and cognition are grounded in the full diversity and richness of human linguistic behavior.

Finally, while the toolkit runs locally rather than relying on remote inference services (i.e., audio is not transmitted to third-party servers), users should be aware that large-scale deployment has an energy footprint that should be weighed against the manual-annotation alternative. Given the impact of data servers on the globe’s environment, fine-tuning and training decisions must be taken cautiously. We invite users to think about these issues critically (Guest et al., 2025).

## Data Availability

All collected data and processed data can be found on our OSF page: https://osf.io/2twqm/overview?view_only=e4d9d6e955084647a94902945b282709

## Code availability

All the code used to create this project is publicly available on our GitHub page: https://github.com/sarmadchandio/tapa.
